# An Automated Method for Quality Control in MRI Systems: Methods and Considerations

**DOI:** 10.3390/jimaging6100111

**Published:** 2020-10-18

**Authors:** Angeliki C. Epistatou, Ioannis A. Tsalafoutas, Konstantinos K. Delibasis

**Affiliations:** 1 Department of Computer Science and Biomedical Informatics, University of Thessaly, 35131 Lamia, Greece; agg_epistatou@yahoo.gr; 2 Occupational Health and Safety Department, Radiation Safety Section, Hamad Medical Corporation, Doha P.O. Box 3050, Qatar; tsalasjohn@yahoo.gr

**Keywords:** MRI quality control, ACR phantom, automated method, randomization, SNR, SNR uniformity, percentage image uniformity, percentage ghosting ratio

## Abstract

Objective: The purpose of this study was to develop an automated method for performing quality control (QC) tests in magnetic resonance imaging (MRI) systems, investigate the effect of different definitions of QC parameters and its sensitivity with respect to variations in regions of interest (ROI) positioning, and validate the reliability of the automated method by comparison with results from manual evaluations. Materials and Methods: Magnetic Resonance imaging MRI used for acceptance and routine QC tests from five MRI systems were selected. All QC tests were performed using the American College of Radiology (ACR) MRI accreditation phantom. The only selection criterion was that in the same QC test, images from two identical sequential sequences should be available. The study was focused on four QC parameters: percent signal ghosting (PSG), percent image uniformity (PIU), signal-to-noise ratio (SNR), and SNR uniformity (SNRU), whose values are calculated using the mean signal and the standard deviation of ROIs defined within the phantom image or in the background. The variability of manual ROIs placement was emulated by the software using random variables that follow appropriate normal distributions. Results: Twenty-one paired sequences were employed. The automated test results for PIU were in good agreement with manual results. However, the PSG values were found to vary depending on the selection of ROIs with respect to the phantom. The values of SNR and SNRU also vary significantly, depending on the combination of the two out of the four standard rectangular ROIs. Furthermore, the methodology used for SNR and SNRU calculation also had significant effect on the results. Conclusions: The automated method standardizes the position of ROIs with respect to the ACR phantom image and allows for reproducible QC results.

## 1. Introduction

Magnetic resonance imaging (MRI) is based on the nuclear magnetic resonance (NMR) physical phenomenon [1]. MRI systems do not use ionizing radiation but strong magnetic fields and radiofrequency electromagnetic radiation (RF pulses). The absence of ionizing radiation risks is the first reason why MRI is an attractive imaging option. The second is that, since the imaging principle of MRI is not based on X-ray attenuation but on the magnetization properties of tissues (relaxation times T1, T2, and T2*), completely different images than those acquired in CT are obtained. MRI provides information for both anatomical and functional characteristics of human tissues and by modifying the acquisition parameters different tissues and functional characteristics can be highlighted or suppressed, facilitating diagnosis.

While the use of MRI does not involve ionizing radiation, this does not mean that it has zero risks, since safety issues do exist [2]. Therefore, the safety culture inherent in the use of X-ray equipment, though of a different form, also exists in the use of MRI. However, there is another risk common to both MRI and X-ray: the risk of misdiagnosis. A misdiagnosis can put the life of a patient in danger, since a wrong diagnosis can delay or hinder the correct treatment, something that may lead even to patient death. While misdiagnosis can be due to a human error (false or incomplete interpretation of the radiologist), it may also be due to the defective operation of the MRI system, which may mislead the radiologist. 

The complexity of the operation of an MRI system and the physicians’ need for sustainable diagnostic performance raised the necessity for applying a quality assurance (QA) program. QA incorporates a series of quality control (QC) tests for checking the MRI system performance stability and detecting any variations of parameters that affect image quality. In answer to this need, the American College of Radiology (ACR) launched a QA program in the US in order to check the compliance of installed MRI systems with quality standards, using the ACR MRI accreditation phantom (henceforth referred to as ACR phantom) [3,4]. More recently, ACR published an updated manual with detailed instructions on QC tests with the ACR phantom [5]. The American Association of Physicists in Medicine (AAPM) published an extensive report on the acceptance testing and quality assurance procedures for MRI facilities [2]. In this AAPM report the ACR phantom was used for all quality control (QC) tests regarding image quality. 

Since image quality is affected by the technical characteristics of each system but also by the selection of the acquisition parameters, the ACR accreditation protocol requires the acquisition of one locator image and four image series using specific sequences and parameters, two of which are the same for all MRI installations, and the other two are the T1 and T2 weighted brain scan series using the MRI site’s acquisition parameters [3,4]. The controlled parameters, the acquisition sequences type (T1 or T2 weighted), the slice number(s) used for the evaluation in each test, and the pass limits are given in Table 1. An eighth QC test is the visual evaluation of all images to confirm the absence of artifacts.

The evaluation of image quality in the acquired MR images is based on simple techniques that can be performed on site using the software of each MRI system or off-site using one of the many commercially available or free viewers compatible with the Digital Imaging and Communications in Medicine (DICOM) format of MRI images. Most of them rely on the measurements of the signal value and noise (mean pixel value (PV) and pixel value standard deviation (PVSD)) of regions of interest (ROIs) defined by the user on selected locations on the images, others require the measurement of distances or visual observation to identify whether some details or artifacts are visible or not. While simple, the analysis is laborious, and quite some time is needed to perform the analysis, record the results, and document the QC outcome. The known problem with intra- and inter-observer variation is also an issue regarding the reproducibility of QC analysis results.

For this reason, many groups have investigated the possibility of performing image analysis procedures in an automated way using in-house made software. To our knowledge, the first successful attempt was made back in 1986 by Covell et al. [6] using an in-house phantom and software for the evaluation of five parameters: signal-to-noise ratio (SNR), spatial distortion, spatial resolution, slice thickness, and slice separation. Barker et al. [7] proposed in 1992 a semi-automated method that was tailored to quantitative analyses, and the phantom used was constructed from bottles containing agarose gels doped with paramagnetic salts for studying T1 and T2. Hyde et al. [8] developed a semi-automated method to assess scanner variability for spatial anisotropy, distortion and resolution, slice thickness and separation, horizontal and vertical voxel size, and SNR imaging parameters, using a rectangular acrylic enclosure phantom containing an array of 60 square acrylic rods. 

Bourel et al. [9] developed a QC protocol and respective software for automatic analysis of images acquired using a very elaborate commercially available QC phantom and reported the results of 3.5 years of weekly routine checks in three MRI scanners. The parameters evaluated were SNR, image uniformity, geometrical distortion, slice thickness, slice profile, and spatial resolution. The authors concluded that the automated method has proved accurate, reliable, fast, and capable of being implemented on a routine basis. Simmons et al. [10] developed an automated analysis procedure for functional MRI imaging (fMRI). A spherical phantom filled with copper (II) sulphate solution placed in a loading annulus was used and daily measurements were designed to measure SNR, signal-to-ghost ratio (SGR), short-term signal drift, and artifacts and study the long-term (day-to-day) stability of these measures. An automated method for QC in fMRI was also proposed by Stöcker et al. [11]. Ihalainen et al. [12] developed automatic image analysis software to objectively determine 11 image quality parameters using Eurospin phantom set images. 

While other studies have also proposed automated and semi-automated methods for evaluating image quality of MRI images using different in-house, custom-made, or commercially available phantoms, there are four more studies that should be mentioned. The first is the study of Gardner et al. [13], where using the phantom that has been used in other studies [6,8], the authors have shown that automated analysis of a test object can reveal image degradation long before trained human observers are able to do so, proving in this way the usefulness of routine QC programs using phantoms. The other three studies are those by Fitzpatrick [14], Davids et al. [15], and Sun et al. [16], where fully automated methods for the analysis of ACR phantom images were developed using MATLAB^TM^ v.2016b (The MathWorks Inc, Natick, MA, USA). Fitzpatrick [14] compared the results obtained with the automated method with those performed manually by human observers. While the automated method’s results were generally well correlated with those of human observers, problems of the automated procedure mainly for the high contrast resolution where reported, as well as problems of intra-observer variability. The Davids et al. [15] method was successful in measuring SNR, image uniformity, ghosting artifacts, chemical shift, spatial resolution, and spatial linearity, but it could not automate the detection of the LC test patterns. The Sun et al. [16] method successfully measured geometric distortion, slice thickness accuracy, slice position accuracy, percentage intensity uniformity (PIU), percentage signal ghosting (PSG), high contrast spatial resolution, and low contrast object detectability, following the AAPM nomenclature for the QC tests [2]. Though details are not given, Sun et al. [16] have used an image of observer visual contrast sensitivity calibration on the monitor used to match human observer results with those of automated method for high contrast and possibly for low contrast object detectability test. 

In this study, software for the automatic evaluation of image quality was developed in MATLAB^TM^ R2013 and was implemented on ACR phantom images acquired in MRI scanners from different manufacturers in the context of acceptance or routine QC tests. These QC tests had been performed according to the Greek MRI QC protocol [17], which is in line with the AAPM protocol [2], with only some minor differences. The evaluation of these images had been previously performed using manual methods, on-site using the MRI systems software or off-site using ImageJ free software (https://imagej.nih.gov/ij/download.html), and therefore the QC results were available for comparison with the results of the automated methods. 

In this study, the results of the automated evaluation of four parameters are reported: percent signal ghosting (PSG), percent image uniformity (PIU), SNR, and SNR uniformity (SNRU). The methodology used is presented in detail as guidance for anyone that wishes to build his/her own program for automated MRI QC. Though similar methods have been previously published on automated MRI QC methods, this study focuses on QC result reproducibility problems, which may arise from variation in the position where ROIs are located with respect to the phantom and on the effect on the SNR calculation that different methods that have been proposed for its calculation have. For this purpose, a randomization method was introduced in the measurement of PSG, using random variables normally distributed around their expected positions, appropriately applied to coordinates of the ROIs and their width and height, relative to the image pixilation, pixel size, and phantom placement. This process emulates the variability induced by the manual placement of ROIs, and provides the ability to perform a great number of test repetitions to obtain statistically significant results when comparing the automated method with the simulated manual placement, something that otherwise would have been almost impossible to achieve. Furthermore, SNR and SNRU were calculated using different approaches, concerning one or two image slices from the same, or two identical, sequences, acquired with minimal intermediate delay. The aforementioned combinations were further combined with different ROI selection. It is worth noting that although the calculation of SNR is described in the National Electrical Manufacturers Association (NEMA) standard in multiple alternative ways [18,19], it has not included the ACR accreditation program QC tests, possibly due to lack of a single, well established and widely accepted method of calculation. The use of more than one MRI system is necessary, since images of different imagers may present varying artifacts around the central image area and close to the image edges and therefore affect the calculation of PSG and underline further the importance of ROIs positioning in its calculation. Regarding the PIU, SNR, and SNRU, the comparisons between alternative definitions will be statistically more significant when applied to multiple imagers, whereas the robustness of the proposed software method will be better assessed.

## 2. Materials and Methods

### 2.1. Brief Phantom Description

The ACR phantom (J.M. Specialty Parts, San Diego, CA, USA) is a hollow cylinder of acrylic plastic closed at both ends. The inside diameter of the phantom is 190 mm, and the inside length is 148 mm. The phantom is filled with a solution of nickel chloride and sodium chloride (10 mM NiCl_2_ and 75 mM NaCl). Inside the phantom, there are various structures designated for measuring geometric accuracy, high-contrast spatial resolution, slice thickness accuracy, slice position accuracy, image intensity uniformity, percent signal ghosting, and low-contrast detectability. The “NOSE” and “CHIN” indications are etched on the phantom’s surface to assure proper positioning of the phantom, as if it were a patient’s head.

### 2.2. Data Acquisition

The standard scanning protocol of the ACR phantom comprises a localizer scan and a sequence of a set of 11-slice, spin-echo, axial T1-weighted images. The first slice is positioned with respect to the two crossed wedges shown in the sagittal localizer image. Although the scan parameters may vary depending on the MRI unit tested and the tester’s preferences, common scanning parameters are Repetition Time (TR) Echo Time (TE), TR/TE = 1000/30 ms; Field of View (FOV) equal to 250 mm; acquisition matrix = 256 × 256 pixels; slice thickness/slice gap = 5 mm/5 mm. Other common choices include TR/TE = 500/20 ms and FOV in the range [220, 256] mm. Regarding bandwidth, a frequency around the middle of the available range is selected. The frequency encoding direction for most tests is from left-to-right (along the image columns). However, during acceptance testing, images with the frequency encoding direction in the anterior–posterior and in the superior–inferior (head–feet) direction (with the phantom base positioned parallel to the MRI table) are also acquired. In the following paragraphs, the theoretical background used to build the QC program functions is presented in brief.

In this work, 21 QC tests were utilized from five (5) different MR imagers. For each QC test, sequence acquisition was repeated with identical settings, within less than 5 min from the first acquisition (herein called series A and B, respectively). Although two acquisitions are not required by the ACR protocol, the acquired series A and B are utilized in this work for a comparison with the SNR and SNRU definition that involves two different slices of the same sequence. 

### 2.3. Theoretical Background and Programming Methodology

For the quantification of image quality, the definition of a number of ROIs is required, with regard to the following tests: percent signal ghosting (PSG), percentage image uniformity (PIU), signal to noise ratio (SNR), and signal to noise ratio uniformity (SNRU). These ROIs are described in detail below for the different QC tests. The first step for the automatic placement of these ROIs is the identification of image pixels that contain the ACR phantom against the background. The relevant algorithm is described in the Appendix A. 

#### 2.3.1. Percent Signal Ghosting (PSG)

**Figure 1:**
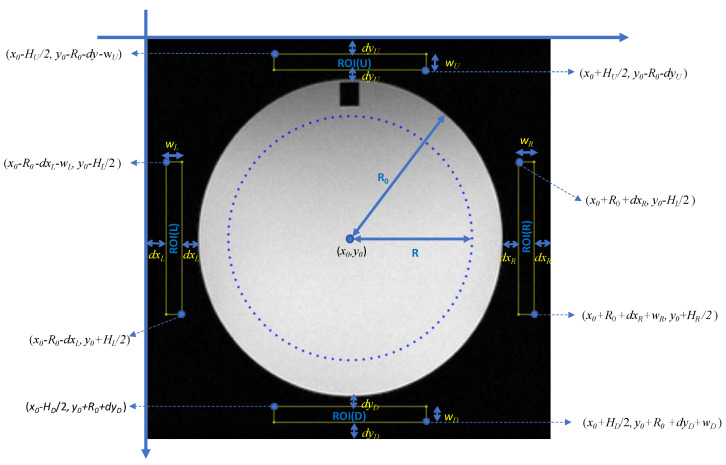
The main circular ROI and the placement of the four rectangular ROIs are shown superimposed on a typical image of slice #7 of the ACR phantom.

For the PSG QC test (also referred to as ghosting ratio), five ROIs are applied to slice #7 of the ACR phantom, as shown in Figure 1. One circular ROI covers the central region of the phantom, and the mean PV of this ROI is defined as the phantom signal intensity $\bar{S}$. Four additional rectangular ROIs, placed up (U), down (D), left (L), and right (R), outside the ACR phantom provide the respective signal intensities in the background of the image, denoted as ${\bar{S}}_{U},{\bar{S}}_{D},\;{\bar{S}}_{L},{\bar{S}}_{R}$, respectively. The PSG is determined using the following equation:

(1) $$PSG\;\left(\%\right)=100\times \left|\frac{\left({\bar{S}}_{R}+{\bar{S}}_{L}\right)-\left({\bar{S}}_{U}+{\bar{S}}_{D}\right)}{2\bar{S}}\right|$$

To pass the test, the ghosting ratio must be less than or equal to 2.5% according to the ACR [4] and 1% according to the AAPM and the Greek MRI QC protocol [2,17].

To automate the placement of the required ROIs and calculate the PSG, the following procedure was used. A main circular ROI, concentric with the phantom, with radius $\;R=0.8\;\times \;R_{0}$ and area approx. 64% of the phantom circle was used to define the useful field of view (UFOV). The dimensions of the four rectangular bar-shaped ROIs are set as the following: the value of the width, *w* (in pixels), is set such that the ROI lies 0.1$\;\times \;R_{0}$ from the edge of the phantom as well as from the border of the image. Thus, the width *w* may be different for each one of the four (4) rectangular ROIs, denoted as *w_R_*, *w_L_*, *w_U_*, and *w_D_*. The long dimension *H* of each ROI is calculated (in pixels), so that the area of the ROI equals to 10 cm^2^. Consequently, *H* may also be different for each one of the four (4) rectangular ROIs, denoted as *H_R_*, *H_L_*, *H_U_*, and *H_D_*. Assuming that the pixel size *p_x_*, *p_y_*, is stored in DICOM’s metadata in mm, the value of *H* is calculated as follows:

(2) $$H_{q}w_{q}=1000mm^{2}\frac{1}{pixelArea}\Rightarrow H_{q}=\frac{1000}{w_{q}p_{x}p_{y}}$$

where *pixelArea,* which equals *p_x_*∙*p_y_*, is defined as the area of each pixel in mm^2^ and the subscript *q* denotes the four possible ROIs, thus *q* may take one of the following values: U, D, R, or L. This definition is in agreement with the ACR phantom test guidance for the PSG test [3]. The aforementioned ROIs are shown in Figure 1 for the image of slice #7 of the ACR phantom.

**Figure 2:**
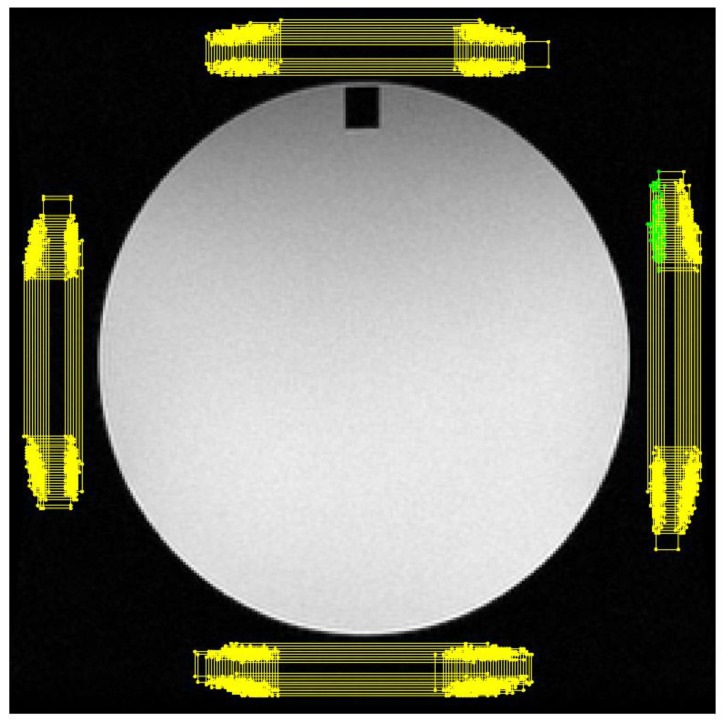
An example of 500 different sets of rectangular ROIs randomly generated to simulate the effect of ROI positioning variation on the PSG test results.

The position and sizes of the four rectangular ROIs that are placed manually vary depending on the dimensions on the FOV, the placement of the phantom within it, as well as due to tester preferences. In order to emulate the variability in the positioning and size of the ROIs, which occurs when a ROI is manually drawn by the tester, and to investigate the effect that this may have on the test results, some randomness in the placement of the rectangular ROIs was introduced. The width (*w_q_*) and the distances of the ROIs from the circular phantom (*dx_q_*,*dy_q_*) were initially set to their standard values, and a random variable that follows the normal distribution with zero mean and standard deviation *σ* = 1 was added. The long dimension *H_q_* for the ROIs was calculated according to Equation (2) using the random values for *w_q_*. Finally, the position of the upper left corner for each ROI was allowed to vary randomly following the zero-mean normal distribution with two possible values for standard deviation *σ* along the long and the short ROI dimension, equal to 1 and 3 pixels, respectively. The different values of the standard deviation *σ* along the long and the short ROI dimension were selected, since the variability along the short dimension is limited by the placement requirements for the ROI. In this way, both the position, the values, and the ratio of dimensions of the bar-like ROIs were allowed to vary without overlapping with the phantom area or crossing the image border. The ratio *H*/*w* was variable and dependent on the available space between the phantom and the image borders, which in turn was dependent on the FOV used for image acquisition. In Figure 2, 500 different ROIs resulting from the above randomization process are shown, resulting in an equal number of PSG values for each image sequence.

#### 2.3.2. Percentage Image Uniformity (PIU)

The percentage intensity uniformity is assessed by identifying and comparing the high and low intensity levels within the phantom region of slice #7. More specifically, the slice #7 is convolved (as denoted by “*” operator) with a binary mask *M* of size (2*r* + 1) × (2*r* + 1) pixels, where $r=\left\lceil \frac{10p_{x}}{\sqrt{\pi }}\right\rceil$ is the radius (in pixels) of a circle with area equal to 1 cm^2^, *p_x_* = *p_y_* defined as above and ⌈x⌉ is the operator that returns the closest integer to *x*, greater than, or equal to *x*. The result is divided by the sum of the non-zero elements *N_M_* of the mask to produce an image *I*_1_:

(3) $$I_{1}=\frac{1}{N_{M}}\left(I*M\right)$$

The positions of two ROIs within image *I*_1_ where the maximum and minimum values ${\bar{S}}_{max},\;{\bar{S}}_{min}$ occur, inside the main circular ROI that defines the UFOV, are determined. The PIU is calculated by the following equation:

(4) $$PIU\%=100\times \left[1-\frac{\left({\bar{S}}_{max}-{\bar{S}}_{min}\right)}{\left({\bar{S}}_{max}+{\bar{S}}_{min}\right)}\right]$$

**Figure 3:**
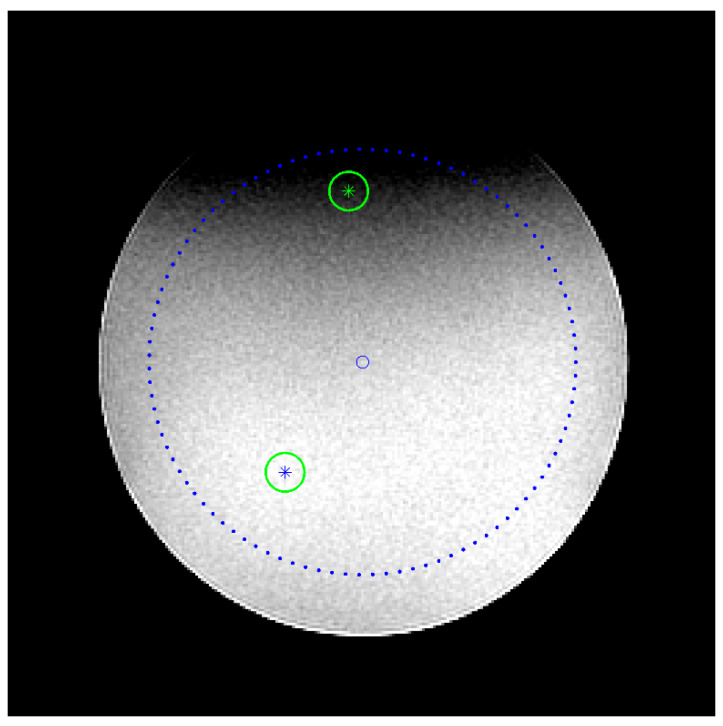
A typical image of the slice #7 of the ACR phantom convolved with mask *M.* The main circular ROI (dashed line) defines the UFOV, and the small circular ROIs are placed around the positions of minimum (ROI up) and maximum (ROI down) signal intensity values. A very small circle is also shown in the center of the phantom.

To pass the test, the value of PIU should be greater than or equal to 87.5% for MRI systems <3T according to the ACR [4] and 90% according to AAPM [2] and the Greek protocol [17]. Figure 3 shows the locations of the ROIs with maximum and minimum signal values for a typical image $I_{1}$, with the small circular ROIs superimposed.

#### 2.3.3. Signal to Noise Ratio (SNR)

The measurement of SNR is probably the most prevalent QC test in MRI, since it is presented in all standards and manuals and extensively studied in many scientific papers [13,14,15,16,17,18,19,20,21,22,23,24]. However, it is not included in the ACR tests [4]. According to NEMA [18], the SNR is calculated using a single image as follows:

(5) $$SNR=\frac{0.665\times \bar{S}}{\sigma_{bkg}}$$

where signal $\bar{S}$ is defined to be the mean signal level in the main circular ROI that covers the UFOV shown in Figure 3, $\sigma_{bkg}$ is the standard deviation of a ROI in the background (air), e.g., anyone of the four rectangular ROIs shown in Figure 1, and the 0.655 factor compensates for the fact that the background signal in magnitude images follows the Rayleigh distribution (a special case of Rician distribution) and not the Gaussian [2,18]. 

An alternative method for measuring SNR is the two-image difference technique, also proposed by NEMA [19]. In this approach, two “identical” images of a homogeneous phantom are acquired with minimal time separation between their acquisition. These images are subtracted, and the SNR is taken to be:

(6) $$SNR=\frac{1.41\times \bar{S}}{\sigma }$$

where $\bar{S}$ is the mean signal in a region of interest (ROI) containing at least the UFOV in both original images (or an average of the two), and σ is the standard deviation of the resulting difference image values inside the same ROI. In this study, we implemented the following variants of SNR automatic calculations:

*A.*  SNR is calculated by Equation (5), applied to a single image (slice #7), using as numerator the mean intensity values of the main circular ROI ($\bar{S}$) and as denominator $\left({\bar{\sigma }}_{bkgd}\right)$ the average of the standard deviations of the left and right rectangular ROI $\left(\sigma_{L}\;and\;\sigma_{R}\right)$, shown in Figure 2 as proposed in the Greek protocol [17]. Therefore, Equation (5) becomes:
  (7) $$SNR=\frac{0.665\times \bar{S}}{\frac{\sigma_{L}+\;\sigma_{R}}{2}}=\frac{2\times 0.665\times \bar{S}}{\sigma_{L}+\;\sigma_{R}}$$

To investigate whether the selection of the background ROI has any effect on the calculation of SNR, Equation (6) was applied four more times, replacing $\frac{\sigma_{L}+\;\sigma_{R}}{2}$ with the appropriate combinations of the SDs of ROIs up, down, left, right, *σ_U_*, *σ_D_*, *σ_L_*, and *σ*_R_, respectively. The four variants of Equation (7) are provided in Table A1 (see Appendix A).

*B.*  According to the Greek MRI protocol [17], the SNR as calculated by Equation (5) can be equally applied to both #7 and #6 homogeneous slices of the phantom acquired during the same sequence. Therefore, two SNR values (SNR_1_ and SNR_2_) are determined (for slices #7 and #6, respectively) using Equation (5). To pass the test, the value of each SNR should be greater than or equal to 80×T, where T is the intensity of the magnetic field in Tesla and the ratio of the two SNRs must be within 0.9 and 1.1, that is:
  (8) $$\;SNR_{1}=\frac{2\times 0.665\times \bar{S_{1}}}{\sigma_{L_{1}}+\sigma_{R_{1}}}$$
  (9) $$\;SNR_{2}=\frac{2\times 0.665\times \bar{S_{2}}}{\sigma_{L_{2}}+\sigma_{R_{2}}}$$
  (10) $$0.9\leq \frac{SNR_{1}}{SNR_{2}}\leq 1.1$$
*C.*  The SNR is calculated by Equation (6), where $\bar{S}$ is the average value of the main circular ROIs (UFOV) of slices #7 (${\bar{S}}_{1}$), #6 (${\bar{S}}_{2}),$ or the average of the two $\left(\frac{\bar{S_{1}}+\bar{S_{2}}}{2}\;\right)$, and *σ_D_* is the standard deviation of the signal intensity of the UFOV of the image produced when subtracting one image from another [2]. In other words, the SNR can be calculated in any of the following ways:
  (11) $$SNR_{1D}=\frac{1.41\times \bar{S_{1}}}{\sigma_{D}}$$
  (12) $$SNR_{2D}=\frac{1.41\times \bar{S_{2}}}{\sigma_{D}}$$
  (13) $$SNR_{12D}=\frac{1.41\times \left(\bar{S_{1}}+\bar{S_{2}}\right)}{2\times \sigma_{D}}\;$$

All the above three definitions of SNR were used in this study.

*D.*  Another variation of the above SNR definitions can be derived, using the subtraction of slice #7 between two identical sequence scans acquired within 5 min or less (already referred to as series A and B, respectively). The concept of two image subtraction is described in the NEMA document [20] and can be applied to images from different acquisitions, provided that 5 min or less have elapsed between the two. Following this concept, Equations (11)–(13) can be used, with the only difference being that the subscripts 1 and 2 are used to describe the signal and noise in slice #7 of the first (series A) and the second acquisition (series B), respectively.

**Figure 4:**
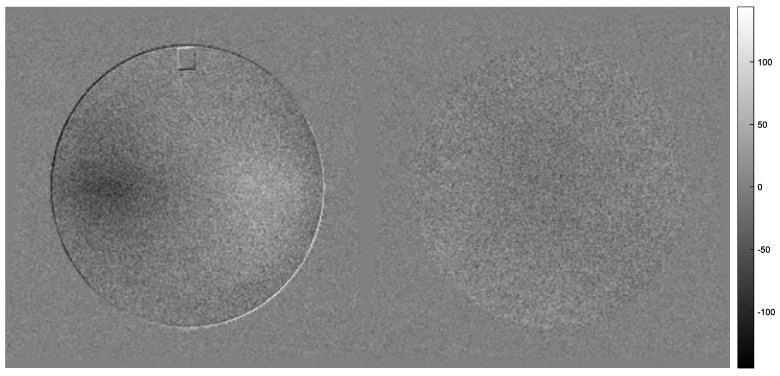
An example of the image that results when subtracting slice #6 from slice #7 acquired in the same acquisition is shown in the left. An example of the image that results when slice #7 acquired in series B is subtracted from slice #7 acquired in series A is shown in the right. Both examples are for sequences acquired with the GE Genesis Signa 1T MRI system.

A typical example of subtracting slice #6 from slice #7 of the ACR phantom from a single sequence, and slice #7 of a series B from slice #7 of series A (as described in the Results section), is shown in Figure 4 (left and right, respectively). In the left of this figure, it can be seen that, since the ACR phantom may not be perfectly aligned along the Z-axis, and slices #6 and #7 may not be centered with respect to each other, the circumference of the phantom is delineated in the first case (left image), something that is not observed in the other case (right image). 

#### 2.3.4. Signal to Noise Ratio Uniformity (SNRU)

**Figure 5:**
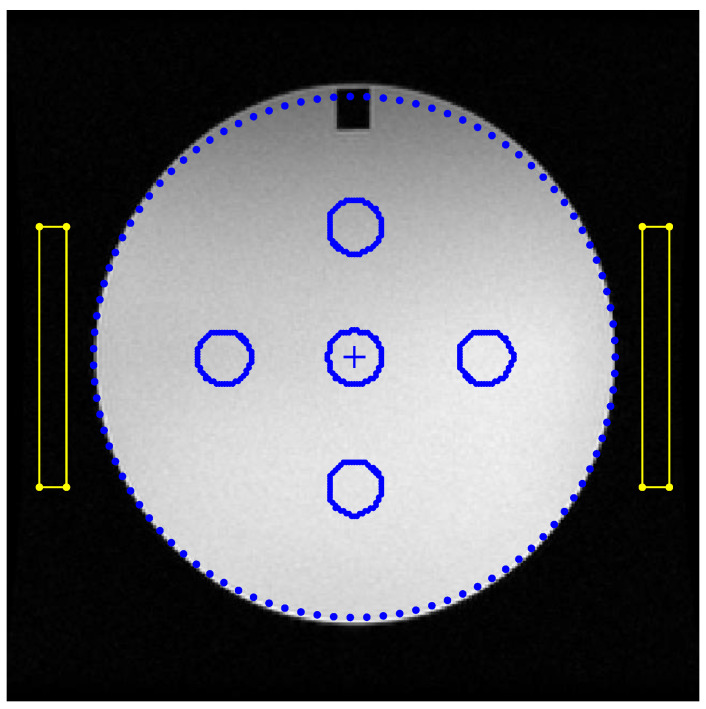
The five circular ROIs inside the phantom and the two rectangular ROIs outside the phantom that are used to calculate SNRU are shown.

The SNRU is a QC check that was first described by Lerski et al. [20], though it was not given this exact name, and was also described by Ihalainen et al. [11] as SNR uniformity. The SNRU is similar to the uniformity QC test made in CT, and it is calculated using five small circular ROIs, one in the center and the remaining ROIs towards the periphery of the phantom at the positions 3, 6, 9, and 12 o’clock, as shown in Figure 5. Although no specific limits exist, the values of 5% (achievable) based on the results of the Ihalainen et al. study [11] and of 10% (maximum acceptable) had been given for reference. The results of the first test (acceptance QC) on a specific MRI system are used as a baseline, and any deviation from the baseline in subsequent tests should be within 10%.

In this study, we positioned a small circular ROI of approximately 1 cm radius at the center of slice #7 of the phantom and four more identical ROIs above, below, right, and left of the central small ROI, at a distance equal to *R*_0_/2, as shown in the Figure 5. The SNR value of each one of the five ROIs is calculated using Equation (7) as follows:

(14) $$SNR_{i}=\frac{2\times 0.65\times {\bar{S}}_{i}}{\sigma_{L}+\sigma_{R}},\;i=1,2,\ldots ,5$$

where $\bar{S_{\iota }}$ (i=1,2,…,5) is the mean values of signal intensity of the five ROIs and $\sigma_{L},\sigma_{R}$ are the standard deviations of the rectangular ROIs in the background. Finally, SNRU is calculated as the percentage ratio:

(15) $$SNRU=100\times \frac{\sigma_{SNR}}{\bar{SNR}}\;$$

where $\sigma_{SNR}$ and $\bar{SNR}$ are the standard deviation and the mean value of the five ROIs’ SNR values. The SNRU can be calculated for slice #6 as well, again using Equation (14) and Equation (15). Furthermore, the ratio of the two SNRUs [SNRU(#7)/SNRU(#6)] can serve as an additional QC index to assess the constancy of SNRU across the Z-axis for the two neighboring slices that are acquired with a small time interval between them. The SNRU ratio should have values in range [0.9, 1.1], which is the same range as given by Equation (10) for the SNR ratio. 

The calculation of SNRU and SNRU ratio can be performed in an alternative way, as described in Section 2.3.3 par. *C*, using the following equations:

(16) $$SNR_{1D,i}=\frac{1.41\times {\bar{S}}_{1,i}}{\sigma_{i,D}}\;$$

(17) $$SNR_{2D,i}=\frac{1.41\times {\bar{S}}_{2,i}}{\sigma_{i,D}}\;$$

where ${\bar{S}}_{1,i\;}$ and ${\bar{S}}_{2,i\;}$ are the signals in each one of the 5 ROIs (*i*=1,…,5) of images #7 and #6, respectively, and $\sigma_{i,D}$ is the standard deviation of the respective five ROIs in the subtraction of slice #6 from slice #7. Finally, Equation (18) is used to calculate *SNRUD*_1_ and *SNRUD*_2_ for slices #7 and #6, respectively,

(18) $$SNRUD=100\times \frac{\sigma_{SNR}}{\bar{SNR}}\;$$

Their ratio is also calculated and checked against the accepted range of [0.9, 1.1]. 

In order to facilitate understanding, Table A1 was added in the Appendix A, summarizing all the variant definitions for SNR and SNRU calculation, along with the relevant symbols, the corresponding text subsections in methodology, and the figures where results of these variants are reported. 

## 3. Results

The QC methodologies described above were applied in images acquired in the context of acceptance or routine QC from five different MRI systems of three different manufacturers (**A-1:** GE Genesis Signa 1T, **A-2:** GE Genesis Signa 1.5T, **A-3**: GE Brivo MR355 1.5T, **B-1:** Philips Panorama HFO 1T, and **C-1:** Siemens Symphony Vision 1.5T). For some systems, images from only one QC were used, while for other systems, images from more than one QC test were utilized. These images were selected from a database of QC images, because two sequential acquisitions of the ACR phantom with identical scanning parameters had been performed in each system the same day and with a time difference of about 5 min (i.e., series A and B), something which was essential for SNR calculations using all methods that have been proposed. 

### 3.1. Percent Signal Ghosting (PSG)

**Figure 6:**
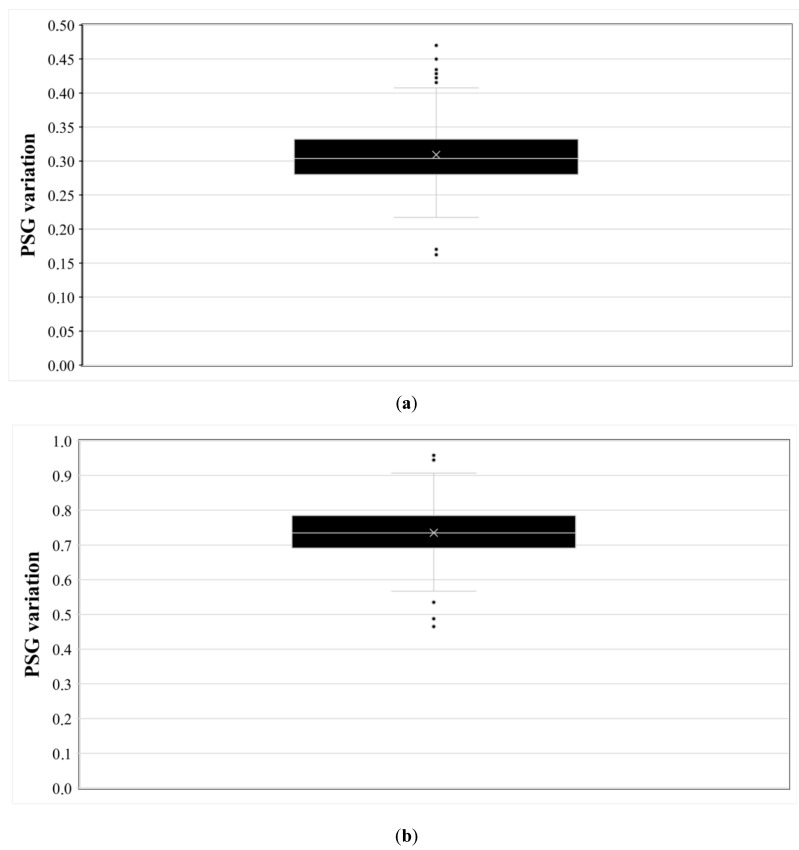
The variation of PSG values depending on the positions of the four rectangular ROIs around the phantom is shown. Mean (x), median (white line within the black boxes), 1st and 3rd quartile (lower and upper black boxes, respectively), SD (whisker bars), and outliers (black data points) are shown. (**a**) A1 (#7 series A), (**b**) B1 (#7 series B).

For the PSG test, 21 images of the slice #7 of the ACR phantom were used (series A). The median value of all images was 0.27%, and the measured values ranged from 0.0% to 0.74%. No image that did not satisfy either the ACR or Greek protocol limit was found. However, when the randomized procedure was applied (resulting to 500 PSG values), it was seen that the PSG values for the same image exhibit a dependence on the positioning of the four rectangular ROIs. An example of this variation is shown in Figure 6a for an image acquired with the GE Genesis Signa 1T (frequency encoding left to right, repetition time TR = 1000 ms, echo time TE = 30 ms, bandwidth BW = 15.63 kHz). The manual QC has given a PSG value of 0.24%, the automatic (without randomization) 0.33%, and the randomized method gave a range of values from 0.16% to 0.47% with a median value of 0.30%. It is worth mentioning that the median value of 0.30% is in good agreement with the deterministic (without randomization) value of 0.33%. No value larger than 0.72% was observed in any of the images, and therefore all images passed the PSV test. 

However, when running the same procedure for series B images (an example of the PSG variation is shown in Figure 6b for #7 of image B1), there was an image from Philips Panorama HFO 1T (frequency encoding left to right, repetition time TR = 1000 ms, echo time TE = 30 ms, BW = 18.6 kHz) where PSG values ranged from 0.47 to 0.96 (median value = 0.74, automatic 0.73) that is close to the pass/fail border. In series B, an image that failed the test with the automatic test was found from Siemens Symphony Vision 1.5T. However, in this case the random PSG calculation did not make much difference, as all values ranged from 1.05 to 1.11. It is notable, however, that in the respective image of series A, the automatically calculated PSG value was only 0.19.

**Figure 7:**
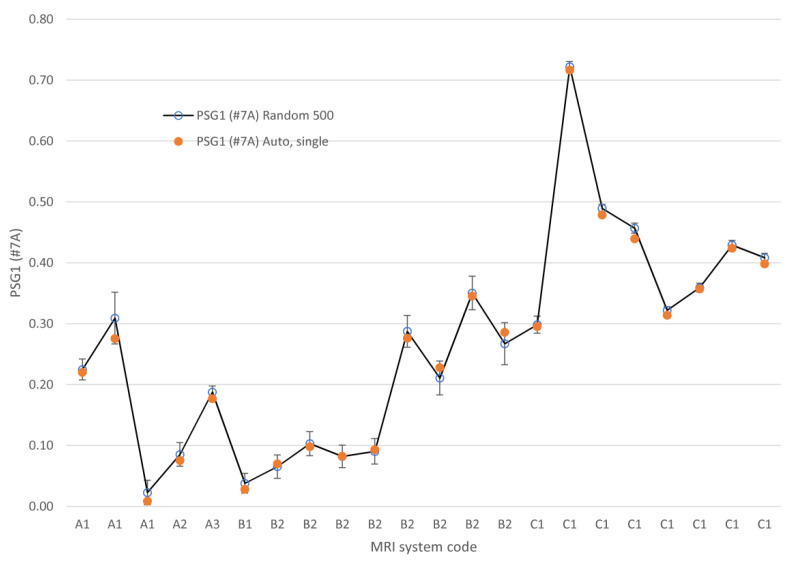
The PSG as calculated by 500 executions of the randomized algorithm with error bars indicating the 99% confidence interval. The value obtained from a single execution of the same automatic algorithm with correct ROI placement.

The PSG as calculated for slice #7 of series A using the automated method with correct ROI placement (without randomization) is shown in Figure 7. The same figure depicts the results of 500 executions of the randomized PSG algorithm with the mean value and the 99% confidence interval. It can be observed that in 18 out of 22 studies the confidence interval includes the result of the single execution. In the rest of the cases, the differences are not excessive.

### 3.2. Percentage Image Uniformity (PIU)

**Figure 8:**
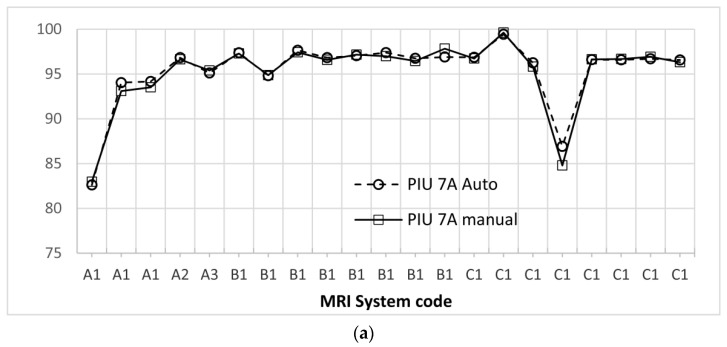

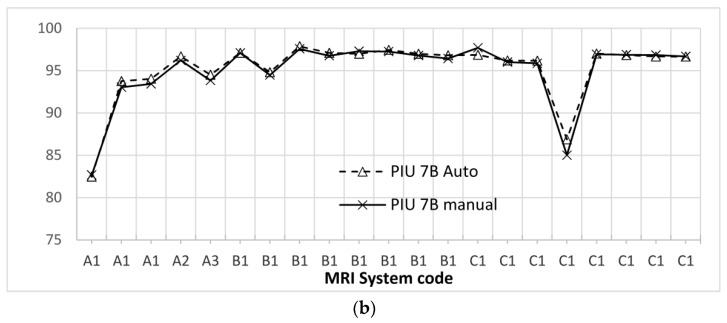
Comparison of the manual and automatic determination of PIU for slice 7 for (**a**) sequence A and (**b**) sequence B for all available studies/ MRI systems.

For the PIU test, the same 42 images of the slice #7 of the ACR phantom were used (21 from series A and 21 from series B). The results from automatic evaluation along with results of the manual evaluations performed by a licensed medical physicist (some of them had been performed in the context of the QCs, and the rest were performed in the context of this study) are shown for comparison in Figure 8a,b. The difference between the automatically and the manually derived values was on average 0.7% (max difference 3.44%) for sequence A and on average 0.5%, (max difference 2.65%) for sequence B.

The median PIU value for all images was 96.7%, and the measured values ranged from 82.5% to 96.7%. Four images failed in this test, and all of them were for the frequency encoding direction head to feet, with the ACR phantom positioned with its flat base parallel to the MRI table. The failure was observed in two images from GE Genesis Signa 1T (one for series A and one for series B) and two from Siemens Symphony Vision 1.5T (one for series A and one for series B). 

### 3.3. Signal to Noise Ratio (SNR)

**Figure 9:**
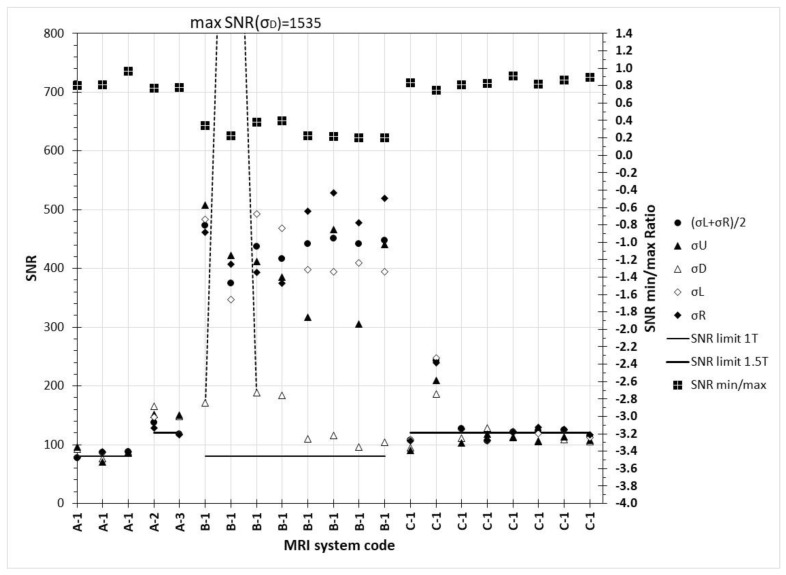
SNR values calculated according to subsection C1 (primary Y-axis) and minimum/maximum SNR value observed (secondary Y-axis). The dashed lines are used to denote that the data point for the second B-1 image for the SNR calculated with σ_D_ in the denominator is off-scale (1535).

Automatic SNR measurements using the standard formula for single image calculation method, using the SD of different ROIs in the background (as described in Section 2.3.3, par. A), had a larger impact on the SNR variability than initially expected, as can be seen in Figure 9. SNR values below the pass limit were observed in 10 out of 21 images, but in only 2 images the test would have failed irrespectively of which ROI was used for the σ of the background. The ratio of minimum to maximum SNR value ratio ranged from 0.2 to 0.97 with a median value of 0.79. The biggest impact of the ROI selection was seen on MRI scanner B (B-1: test#2), which is an open type MRI system. For all other SNR tests in scanner B except this one, the selection of σ_D_ gave the smallest SNR values, and min/max SNR ratios were in the range 0.2–0.39. For B-1: test#2, however, it gave the largest SNR value observed overall (the off-scale value), which also was the only sequence acquired in this system with encoding frequency in the Head-Feet (HF) axis.

**Figure 10:**
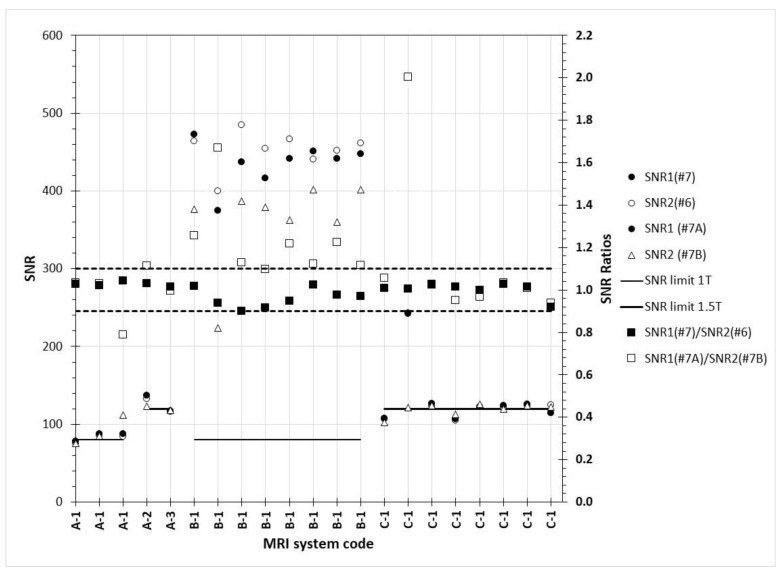
SNR values (primary Y-axis) and SNR_1_/SNR_2_ ratios (secondary Y-axis) calculated according to Section 2.3.3 par. B. SNR_1_(#7) and SNR_1_(#7A) are the same data points, since image #7 is the same with image #7A (different name is used to denote different methodology). The dashed lines are used to denote the lower and upper limits for SNR ratios (secondary axis).

In Figure 10, the SNR values calculated according to Section 2.3.3 par. B, for slices #7 and #6 of the same acquisition, and for slices #7A (=#7) and #7B of different but sequential acquisitions and of their ratios are presented. When referring to the SNR values defined from images #7 and #6 of the same acquisition, it can be seen that in all cases the SNR_1_ and SNR_2_ values were very close to each other, and as a result their ratio SNR_1_(#7)/SNR_2_(#6) was very close to 1. However, for many cases where the images of slice #7 from identical sequential acquisitions were used, the SNR_1_(#7A)/SNR_2_(#7B) ratio was either below 0.9 (3rd A-1 data point) or above 1.1 (all B-1 tests and 2nd C-1 data point).

**Figure 11:**
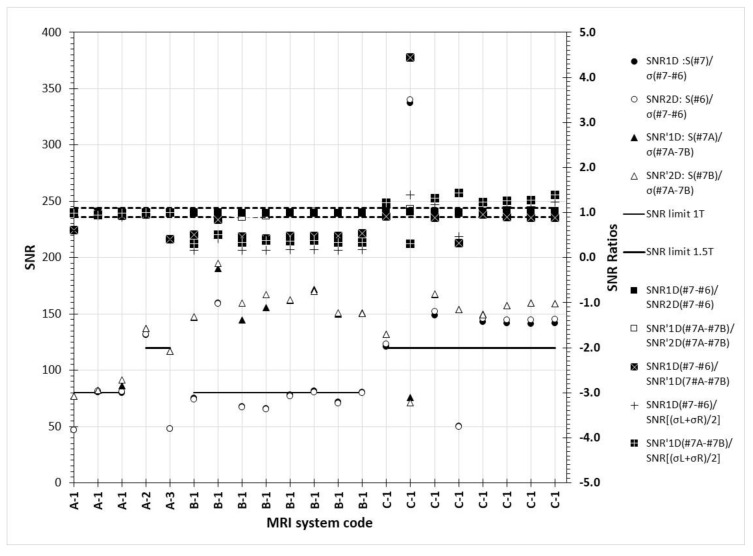
SNR_D1_ and SNR_D2_ values (primary Y-axis) and SNR_D1_/SNR_D2_ ratios (secondary Y-axis) calculated according to Section 2.3.3 par. C & par. D. In the parenthesis, the operation and the images used to derive the SNR value are given. The dashed lines are used to denote the lower and upper limits for SNR ratios (secondary axis).

In Figure 11, it can be seen that in the SNR_D_ values calculated according to Section 2.3.3 par. C, very small differences occur when slices #7 and #6 of the same acquisition are used, and image #6 is subtracted from #7 using in the nominator the signal from #7, #6, or their average. The same was true for the SNR_1D_/SNR_2D_ ratio when slice #7 images from different but identical and sequential acquisitions were used. Additionally, in Figure 11 it can be seen that the ratio of SNR_1D_ (#7–#6)/ SNR’_1D_ (#7A–#7B) was smaller than 1, with one exception where it was much larger (4.44). Most important though is that the ratios SNR_1D_ (#7–#6) and SNR’_1D_ (#7A–#7B) to the SNR calculated according to Equation (7) were most of the times different than one. This means that the two basic methods proposed for SNR calculation give in most cases very different values, something that has been also observed in the manual calculations where both of these two methods were routinely used. 

**Figure 12:**
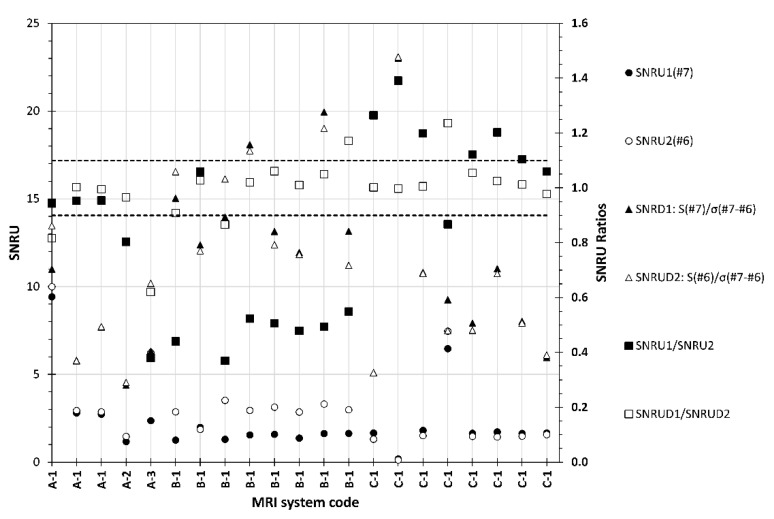
SNRU_1_/SNRU_2_ and SNRDU1/SNRDU2 ratios calculated according to subsection D. In the parenthesis the operation and the images used to derive the SNRU value are given. The dashed lines are used to denote the lower and upper limits for SNRU ratios.

In Figure 12, the SNRU values are given, as derived using the single-image and two-image methods (single acquisition) to calculate initially the SNR and *σ* (SD) values for the central and the peripheral ROI. It can be seen that the two methods for SNR calculation produce different SNRU as well. The two-image method gives constantly larger SNRU values but gives more consistent results compared to the single-image method, as seen from the SNRU_1_/SNRU_2_ and SNRUD_12_/SNRUD_21_ ratio. With the first method, only two data points for SNRU_1_ and three data points for SNRU_2_ are above “achievable” but below “maximum accepted” reference values, whereas with the second method, with one exception only (two data point for A-2), the remaining 40 points are above either of these reference values. 

## 4. Discussion

In this study, software for automatic evaluation of image quality in MRI systems was built, evaluating four QC parameters, PSG, PIU, SNR, and SNRU. Calculations of the PSG were performed using a randomization process applied to several aspects of placement of the four rectangular ROIs, using appropriate statistical distributions, thus simulating the variability of manual placement. Visual inspection of the resulting random ROIs shows that they correlate plausibly with typical actual manual placement. A large number of repetitions of the measurements revealed that the automatically derived PSG values were in good agreement with manually acquired values, considering the large effect that the positioning of the ROIs in the background may have. It was seen that in an image of series B, where the automatic PSG result was 0.73, PSG reached 0.96 when the background ROIs were placed randomly around their expected position used in the automatic test. Though in this case the limit was not exceeded, the fact that the largest PSG value was increased by more than 30% compared to the respective PSG value for the normal ROI positions suggests that there may be cases where the positioning of ROIs can make the difference between pass and fail.

The remaining PIU, SNR, and SNRU parameters were calculated according to their standard definitions as well as to certain definition variants that have been proposed in the literature and are being investigated in this work. More specifically, concerning the PIU test, the measurement method seems quite straightforward, but there is a subtle detail that should be stressed. In the software presented in this study the ROIs are not set around the minimum and maximum pixel value found within the field of view as Davids et al. [16] have proposed, but in the position where the average signal within the ROI is minimum and maximum, respectively. This is in better agreement with the manual/visual method where the tester searches for the darker and brightest areas in the image but the exact position that gives the minimum and maximum average signal requires some trial and error. Another approach is that of Fu et al. [21], where it was proposed the use of a histogram to determine the 5th percentile of the high and low signal values of the whole ACR phantom volume, to serve as *S_max_* and *S_min_*, respectively, in Equation (14).

Concerning the SNR tests, significant differences in the calculated values were observed, depending on the background ROI that was selected for the noise in the one-image method. In the same method, the use of image #6 instead of #7 produced similar SNR results, and the SNR_1_/SNR_2_ ratios were all close to 1, with only one exception. However, it was rather odd that the SNR values were varying when two different but identical sequential acquisitions (series A, B) were acquired, and as a result, in many cases the SNR_1_/SNR_2_ was outside the [0.9, 1.1] range, something that could suggest a temporary drift from the normal operation of the MRI systems or that this SNR method is not reliable.

In the AAPM protocol [2] and in almost all relevant publications dealing with the image subtraction method for the calculation of SNR, it is implied that a single scan is performed and the image subtraction method is applied as described in Section 2.3.3 par. B, using slices #6 and #7 of the same acquisition. In fact, in the recent ACR manual [5], it is explicitly stated (in page 100) that in the two-image method “the two images should be acquired during the same imaging session with a minimal time interval between”. However, on page 91, it is reported that the original two-image method as described in NEMA [19] and applied by Firbank et al. [22] refers to two different acquisitions that are sequentially performed with exactly the same acquisition settings. Firbank et al. [22] reported that on an average of 15 repeated acquisitions, the single-slice method resulted in slightly lower SNR compared to the two-image method (80.6 ± 1.45 *versus* 84.8 ± 1.42) and concluded that the single-image method could be used instead of the two-image method. The single acquisition method is simpler and quicker, but if the two-image method is used, the fact that slices #6 and #7 are not identical should be borne in mind. It should also be noted that both Davids et al. [15] and Fu et al. [21] have used slightly different equations for calculating SNR. The truth about SNR calculations is probably best told in the recent ACR manual [5], where it is clarified that both single and two-image methods are estimates and not rigorous measurements of the true SNR (page 92). 

In our study, in the context of revisiting the various methods used to define SNR, we applied the two-image method both using one sequence and two identical sequences performed one after the other, in agreement with the NEMA rationale [19]. In this way, the same sections of the image are compared, which may be an advantage considering that slices #6 and #7 are at a different distance from the center of the magnet bore and adjoin to different phantom structures. Indeed, Sun et al. [16] reported that slice #6 had on average lower, though not statistically significant, PIU compared to that of slice #7 (91.7 ± 1.45% *versus* 92.5 ± 0.9%). Furthermore, any possible drifts are expected to be accentuated in two sequential but identical acquisitions, provided that no phantom repositioning is made between the two scans. 

When the two-image method was used, it was seen that it does not make much difference which image is subtracted from which, and whether the signal of the first, the second, or their average is used for the SNR calculations. In the two-image method, the use of images from sequential acquisitions (series A and B) gave very different SNR values, both smaller and larger compared to those obtained with the two-image but single acquisition method. This finding is surprising and hard to explain, given the fact that these methods are supposedly interchangeable. 

Concerning the SNRU tests, the results obtained using the single-image or the two-image method vary significantly. This test resembles the uniformity test made in CT scanners, and though it is not used in most QC protocols found in the international literature, it provides useful information about the signal uniformity in the successive uniform slices of the ACR phantom. 

The proposed software system can be used for performing the routine QC tests on clinical MR imagers. The automated process facilitates fast and accurate QC test performance, removing the human factor that often induces unnecessary variability in the results. The available alternative definitions in certain QC parameters that have been implemented provide the user with the ability to compare them in his/her own settings and thus decide which one to utilize. This may be proven particularly useful in cases where one QC parameter value is marginally close to the pass/fail threshold.

## 5. Conclusions

The main findings of this study are that PSG calculation is affected by the position of the ROIs in the background that potentially can make the difference between pass or fail, and that SNR and SNRU are parameters that greatly vary depending on the method used to derive them. Single-image and two-image methods and their variants tested in this study produced large variations. Unlike what we expected, the same calculations on a pair of images from the same and from different acquisitions (with a time interval of about 5 min from each other) sometimes give significantly different results, something which could not be explained. 

More data are required in order to decide whether these results are attributed to acquisition errors (e.g., phantom movement, temperature differences), MRI scanner drifts, or some other errors in the application of these QC methods. Since automatic evaluation enables the calculation of all QC parameters and their variants without extra effort by the tester, the routine acquisition of two identical sequential acquisitions of the ACR phantom instead of one and the routine calculation of all QC parameter variants may give answers with regard to which variant (if any) is more sensitive than the other in detecting performance drifts during long-term performance monitoring of MRI systems.

## Figures and Tables

**Table 1:** USA accreditation program QC tests.

QC Test	Series	Slice No	Limits
1. Geometric accuracy	T1	Localizer, #1, #5	≤± 2 mm
2. High-contrast spatial resolution	T1	#1	≤ 1 mm
3. Slice thickness accuracy	T1, T2	#1	5 mm ± 0.7 mm
4. Slice position accuracy	T1, T2	#1 & #11	≤±5 mm (≤±4 mm)
5. Image intensity uniformity	T1, T2	#7	≥87.5% (<3T), ≥82% (3T)
6. Percent-signal ghosting	T1	#7	≤0.025
7. Low-contrast object detectability	T1, T2	#8-#11	≥9 (<3T), ≥37 (3T)

**Figure A1:**
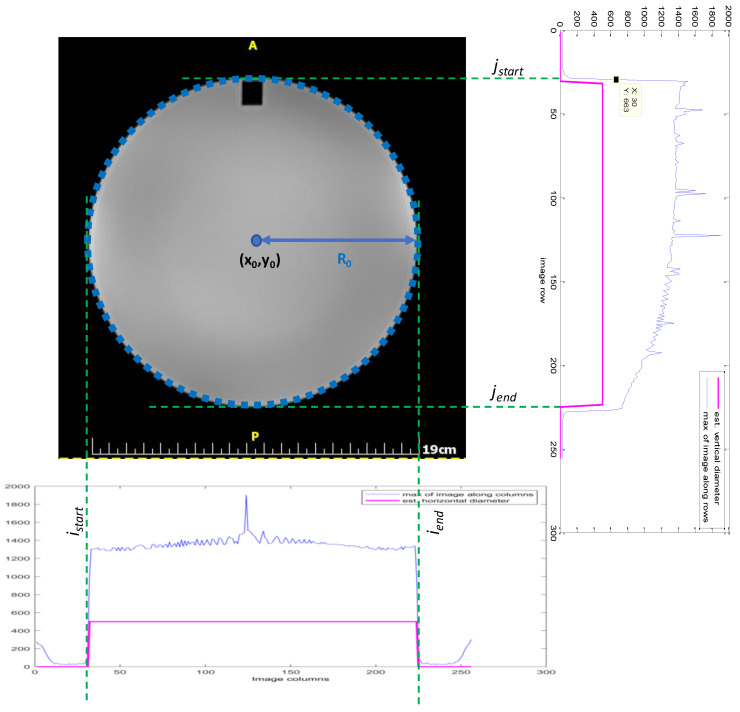
An example of automatic detection of the circular ACR phantom.

## Appendix A

### Automatic Detection of Circular Phantom: Determination of the Center and Radius of the Phantom Fitting Circle

By convention, the intrinsic image x-y coordinate system starts from the upper left corner (x = 0; y = 0) and image columns are defined across the x-axis (Right–Left—RL-direction), while image rows are defined across the y-axis (Anterior–Posterior—AP- or Head–Feet—HF-direction). Let $P_{x}$ be the intensity profiles parallel to the x-axis that cross the $N_{c}$ different columns and *P_y_* be the intensity profiles parallel to the y-axis that cross the $N_{r}$ different rows. More formally, the x-profile *P_x_* of any row *j* is a function of column *i* and the y-profile *P_y_* of any column *i* is a function of row *j*, defined as follows:

(19) $$P_{x,j}\left(i\right)=S\left(i,j\right),\;j=1,2,3,\ldots \;,\;N_{r}$$

(20) $$P_{y,i}\left(j\right)=S\left(i,j\right),\;i=1,2,3,\ldots \;,\;N_{c}$$

where S(i,j) is the signal intensity (i.e., the pixel value, PV) of each pixel with coordinates (*i*,*j*) = (*x*,*y*), and $N_{r}$ and $N_{c}$ are the number of rows and columns of the image matrix, respectively. MRI images are usually acquired using a matrix of dimensions *N_r_*× *N_c_* = 256 × 256.

Now let $S_{max}$ be the maximum signal intensity (S), i.e., the maximum PV of all the pixels of the image. For the $P_{x,j}$ profile of any row *j*, let $\left(i_{1},j\right)$ and $\left(i_{2},j\right)$ be the first and the last pixels in this row *j* for which the following constraint is satisfied:

(21) $$S\left(i_{1},\;j\right)\geq 0.25\times S_{max}\;AND\;S\left(i_{2},j\right)<0.25\times S_{max}$$

Likewise, for the $P_{y,i}$ profile of any line *i*, let $\left(i,j_{1}\right)$ and ($i,j_{2})$ be the first and the last pixel of this column *i* for which the following constraint is satisfied:

(22) $$S\left(i,\;j_{1}\right)\geq 0.25\times S_{max}\;AND\;S\left(i,j_{2}\right)<0.25\times S_{max}$$

The value of $0.25\times S_{max}$ is used as an arbitrary threshold in order to detect pixels with intensity significantly higher than the background (i.e., air and noise), which consequently are pixels of the imaged phantom.

The image line that passes through the center of the circular phantom is determined as the line *j* for which the expression *i*_2_-*i*_1_ is maximum. Let us define for this specific profile $P_{x}\;$, $i_{start\;}=i_{1},$ and $i_{end}=i_{2}$. Similarly, the image column that passes through the center of the circular phantom is determined as the column *i* for which the expression *j*_2_-*j*_1_ is maximum. Let us define for this specific profile $P_{y}\;$, $j_{start\;}=j_{1}$, and $j_{end}=j_{2}$.

Then the coordinates $(x_{0},\;y_{0}$) of the center of the circle are easily obtained as:

(23) $$x_{0}=\frac{i_{start}+i_{end}}{2}$$

(24) $$y_{0}=\frac{j_{start}+j_{end}}{2}$$

and the radius of the phantom *R*_0_ is calculated as:

(25) $$R_{0}=min\left(\frac{i_{end}-i_{start}+1}{2},\frac{j_{end}-j_{start}+1}{2}\right)$$

In Figure A1, the MRI image of a slice # 7 of the ACR phantom is shown, along with the $P_{x}$ and $P_{y}$ intensity profiles, along the line and column that pass through the center of the circular phantom. The binary (purple) line in the horizontal and vertical graphs of Figure A1 indicates the calculation of *i_start_*, *i_end_* and *j_start_*, *j_end_*, respectively. The circle that is defined according to the procedure described above is shown superimposed on the image of the phantom, as well as the circle’s center and radius defined by Equation (23,24), and by Equation (25), respectively.

**Table A1:** Definition of SNR and SNRU parameters and their variants, equations and symbols used, and figures, where the results of these parameters are reported.

QC Test Parameter	1 or 2 Images	1 or 2 Sequences	ACR Image Slice No.	Subsection No. and Basic Equation	Variants Used (S Denotes Average Signal and σ the Standard Deviation)	Figure No
SNR	1	1	#7	Subsection C1, $SNR=\frac{0.665\times \bar{S}}{\sigma_{bkg}}$ (5)	$SNR=\frac{2\times 0.665\times \bar{S}}{\sigma_{L}+\;\sigma_{R}}$ (7), $SNR=\frac{0.665\times \bar{S}}{\sigma_{U}},\;SNR=\frac{0.665\times \bar{S}}{\sigma_{D}}$, $SNR=\frac{0.665\times \bar{S}}{\sigma_{R}}$, $SNR=\frac{0.665\times \bar{S}}{\sigma_{L}}$	9
	2	1	#7 & #6	Section 2.3.3 par. C, $SNR=\frac{1.41\times \bar{S}}{\sigma }$ (6)	$SNR_{1D}=\frac{1.41\times \bar{S_{1}}}{\sigma_{D}}$ (11) Subscript 1 refers to i.e., S(#7) and σ_D_ refers to the subtraction image i.e., σ(#7-#6)	10
		1	#7 & #6		$SNR_{2D}=\frac{1.41\times \bar{S_{2}}}{\sigma_{D}}$ (12) Subscript 2 refers to #6 i.e., S(#7). σ_D_ refers to the subtraction image (#7-#6) i.e., σ (#7-#6)	-
		1	#7 & #6		$SNR_{12D}=\frac{1.41\times \left(\bar{S_{1}}+\bar{S_{2}}\right)}{2\times \sigma_{D}}$ (13) Subscript 1 refers to #7, and subscript 2 refers to #7 and σ_D_ refers to the subtraction image (#7-#6) i.e., σ(#7-#6)	-
		2	#7A & #7B	Section 2.3.3 par. D, $SNR=\frac{1.41\times \bar{S}}{\sigma }$ (6)	$SNR_{1D}^{’}=\frac{1.41\times \bar{S_{1}}}{\sigma_{D}}$ (11) Subscript 1 refers to #7, i.e., S(#7A) and σ_D_ refers to the subtraction image σ(#7A-#7B). The ’ is used to denote that two different series A & Β are used	11
SNR ratio (SNR_1_/SNR_2_)	1	1 (2)	#7 & #6 or 7A & 7B	Section 2.3.3 par. B, $SNR=\frac{0.665\times \bar{S}}{\sigma_{bkg}}$ (5)	$\;SNR_{1}=\frac{2\times 0.665\times \bar{S_{1}}}{\sigma_{L_{1}}+\sigma_{R_{1}}}$ (8), $SNR_{2}=\frac{2\times 0.665\times \bar{S_{2}}}{\sigma_{L_{2}}+\sigma_{R_{2}}}$ (9), $0.9\leq \frac{SNR_{1}}{SNR_{2}}\leq 1.1$ (10) Subscript 1 refers to slice #7 (or #7A) and subscript 2 refers to slice #6 (or #7B)	10
	1	2	#7 & #6	Section 2.3.3 par. C, $SNR=\frac{1.41\times \bar{S}}{\sigma }$ (6)	$SNR_{1D}^{}=\frac{1.41\times \bar{S_{1}}}{\sigma_{D}}$ (11), $\;SNR_{2D}^{}=\frac{1.41\times \bar{S_{2}}}{\sigma_{D}}$ (12), $\;0.9\leq \frac{SNR_{1D}^{}}{SNR_{2D}^{}}\leq 1.1$ (13) Subscript 1 refers to #7, subscript 2 refers to image #6, and σ_D_ refers to the subtraction image (#7–#6).	11
	2	2	#7A & #7B	Section 2.3.3 par. D, $SNR=\frac{1.41\times \bar{S}}{\sigma }$ (6)	$SNR_{1D}^{’}=\frac{1.41\times \bar{S_{1}}}{\sigma_{D}}\;$ (11), $\;SNR_{2D}^{’}=\frac{1.41\times \bar{S_{2}}}{\sigma_{D}}$ (12),$\;0.9\leq \frac{SNR_{1D}^{’}}{SNR_{2D}^{’}}\leq 1.1$ (13) Subscript 1 refers to #7A, subscript 2 refers to image #7B and σ_D_ refers to the subtraction image (#7A–#7B). The ’ is used to denote that two different series A & Β are used.	11
SNRU	1	1	#7	$SNRU=100\times \frac{\sigma_{SNR}}{\bar{SNR}\;\;\;}$ (15) where $\sigma_{SNR}$ and $\bar{SNR}$ are the standard deviation and the mean value of the 5 ROIs’ SNR values	$SNR_{i}=\frac{2\times 0.65\times {\bar{S}}_{i}}{\sigma_{L}+\sigma_{R}},\;i=1,2,\ldots ,5$ (14)	12
SNRUD	2	1	#7 & #6		$SNR_{1D,i}=\frac{1.41\times {\bar{S}}_{1,i}}{\sigma_{i,D}}$ (16), $SNR_{2D,i}=\frac{1.41\times {\bar{S}}_{2,i}}{\sigma_{i,D}}$ (17), i=1,2,…,5 Subscript 1 refers to image #7, subscript 2 refers to image #6	
SNRU ratio	2	1	#7 & #6	SNRU_1_/SNRU_2_	SNRU(#7)/SNRU(#6) with SNRU values calculated using Equation (14)	
SNRUD ratio	2	1	#7 & #6	SNRUD_1_/SNRUD_2_	SNRUD(#7)/SNRUD(#6) with SNRUD values calculated using Equation (16,17)	
